# Dental Law in the District of Columbia

**Published:** 1892-08

**Authors:** 


### DENTAL LAW IN THE DISTRICT OF COLUMBIA.

We congratulate the members of the dental profession in the District of Columbia, that, after years of effort, they have succeeded in securing from Congress a law regulating practice in dentistry.

Charlatanry was more rampant there from the fact that all the surrounding States have had laws, while this small section of government land has been left to the tender mercy of the national legislature. The difficulty of moving so large a body can be appreciated, but, at last, it has been accomplished.

The men who have the control as a Board of Examiners can be depended on to carry out its provisions with vigor and justice.

We desire to express special congratulations that a sensible law has been enacted. The framers have recognized in it that colleges have some rights, and refused to be tempted by the cry of *re-examination.* The law says "all graduates of dental colleges which require a three years' course of study shall be entitled to certificates upon payment of the certificate fee, and *without examination* as to their qualifications." We trust that this reasonable clause may carry force and influence into regions less liberal, and lead to a change of law to a more fraternal basis.

